# The Age-Dependent Response of Carbon Coordination in the Organs of *Pinus yunnanensis* Seedlings Under Shade Stress

**DOI:** 10.3390/plants14172679

**Published:** 2025-08-27

**Authors:** Juncheng Han, Yuanxi Liu, Wenhao Zhang, Guihe Duan, Jialan Chen, Weisong Zhu, Junwen Wu

**Affiliations:** College of Forestry, Southwest Forestry University, Kunming 650224, China; 18988443741@163.com (J.H.);

**Keywords:** *Pinus yunnanensis*, shade stress, age-dependent response, carbon allocation, coordination strategy

## Abstract

To elucidate shade adaptation mechanisms in *Pinus yunnanensis* seedlings across different ages, this study established five light gradients (100% full sunlight as control or CK, 80% as L1, 45% as L2, 30% as L3, and 5% as L4) for experimental treatments on one- and three-year-old seedlings. By analyzing dynamic changes in non-structural carbohydrates (NSCs) and their components within needles, stems, and roots—combined with a phenotypic plasticity assessment, a correlation analysis, and a principal component analysis—we explored the carbon metabolic adaptations under shade stress. The key results demonstrate the following: (1) Increasing shade intensity significantly reduced the NSCs in the needles and roots of both age groups. The stem NSCs markedly decreased under L1 and L2, indicating “carbon limitation.” However, under severe shade (L3 and L4), the stem NSCs stabilized while the stem soluble sugars gradually increased. In three-year-old *Pinus yunnanensis* seedlings under the L3 treatment, the ratio of soluble sugars to starch in the stems reached as high as 5.772 g·kg^−1^, yet the stem NSC content showed no significant change. This pattern exhibited “growth stagnation-carbon enrichment” characteristics. This reveals a physiological strategy for maintaining stem carbon homeostasis through a “structure–metabolism” trade-off under carbon limitation. (2) Shade adaptations diverged by age: one-year-old seedlings employed a short-term “needle–root source–sink reallocation” strategy, whereas three-year-old seedlings developed a “root–stem–needle closed-loop homeostasis regulation” mechanism. (3) Age-specific shade thresholds were identified: one-year-old seedlings required >80% full light to maintain a carbon balance, while three-year-old seedlings exhibited enhanced root carbon storage under moderate shade (45–80% full light). This study clarifies the physiological mechanisms by which *P. yunnanensis* seedlings of varying ages optimize shade adaptation through organ-specific carbon allocation, providing a theoretical foundation for shade management in artificial forests and understory seedling conservation.

## 1. Introduction

Light, as the energy source for plant photosynthesis and an essential environmental factor for growth and development, significantly influences plant morphogenesis [1,2], physiological metabolism [3,4], and population regeneration processes [5]. At the metabolic level, light-dependent regulation (Cytochrome P450 activity) is intrinsically linked to stress responses. Light signals (spectral composition, intensity, photoperiod) modulate *CYP450* activity to influence processes, including secondary metabolite biosynthesis, hormone metabolism, and photoprotective compound production—critical adaptations against pathogen invasion, high-intensity light, and UV stress. Conversely, stress responses reciprocally impact light-dependent metabolism through signaling pathway crosstalk, disrupted light signal transduction, and hormone metabolic reprogramming [6]. This bidirectional coordination maintains a dynamic equilibrium between growth and defense in complex environments. Insufficient light reduces light capture and photosynthetic rates, leading to inadequate biomass accumulation. It also constrains branching resources while prioritizing allocation to aboveground growth [7,8]. Vegetation hydrodynamic models reveal that seedlings in dense plantations maintain stability through a “carbon–hydrodynamic trade-off”: when the xylem water potential falls below a critical threshold, carbon is preferentially allocated to the root vascular tissues rather than aboveground branching [9]. This resource reallocation pattern revises the conventional view of “priority allocation to aboveground parts,” underscoring the dominant role of hydraulic constraints in shade adaptation and integrating the idea that hydraulically constrained carbon transport will guide understory light management. The absorption of light, its availability, and the efficiency of converting absorbed light into biomass constitute the primary factors governing tree and stand growth [10]. Seedling adaptation to light directly determines species population regeneration and the restoration of degraded forest ecosystems [11]. Thus, elucidating seedling adaptation strategies to varying light conditions is crucial for forest vegetation restoration and rehabilitation [12]. In forest ecosystems, light penetrates through the canopy or gaps to reach the understory. Plants regulate their canopy architecture and leaf orientation via multi-spectral polarization reflectance to optimize light capture efficiency and drive morphogenesis—for instance, the blue-to-red (B:R) ratio modulates the leaf inclination angle through polarization properties, enhancing the light utilization efficiency in shaded environments [13]. Consequently, such spectral composition-driven adaptations of the canopy structure govern the spatiotemporal allocation of light resources within plant communities. Through long-term evolutionary processes, plants have developed complex morphological and functional traits that enable them to tolerate environmental stress caused by suboptimal light intensities [14]. By dynamically balancing morphological plasticity and physiological regulation, plants optimize their survival and reproductive strategies in variable light environments. Studies have indicated that moderate shading exerts positive effects on plant growth, development, and morphogenesis, partly by increasing the chlorophyll content, while also effectively mitigating photoinhibition under high-light stress. However, under excessive shading where the light intensity persistently falls below the compensation point, the respiratory consumption exceeds carbon assimilation. This leads to leaf chlorosis and abscission [15], resulting in slender stems, a reduced root biomass [16], a compromised structural stability, and impaired reproductive development. Consequently, optimal light conditions constitute an essential prerequisite for ensuring normal plant growth.

Non-structural carbohydrates (NSCs), primarily composed of soluble sugars and starch, represent direct photosynthetic products that serve as the core carbon reservoir and energy pool for plant respiratory maintenance, growth, and stress adaptation [17], while also providing essential carbon backbones for the emergence [18] and development of new organs (buds, leaves, and roots) during nighttime or overcast conditions [19]. Research demonstrates that, under shade conditions, reduced light harvesting suppresses photosynthesis and constrains NSC synthesis, triggering adaptive carbon reallocation strategies to mitigate energy deprivation [20,21]. During brief shading events, elevated NSC levels—resulting from delayed starch-to-soluble-sugar conversion—provide emergency carbon reserves. Conversely, prolonged low irradiance modulates the NSC mobilization efficiency across organs through the m6A RNA modification-mediated post-transcriptional regulation of carbohydrate metabolic genes [22]. This post-transcriptional control enables rapid responses to fluctuating carbon demands, preventing “carbon starvation” risks during droughts and other stressors [23]. Furthermore, the NSC levels and dynamics regulate plant carbon balance strategies, resource allocation patterns, adaptability, and resilience in the face of environmental change [24,25].

*Pinus yunnanensis*, an endemic coniferous species in southwestern China, exhibits an exceptional drought tolerance and adaptability. It serves as a keystone species for ecological restoration and carbon sequestration initiatives [26]. However, climate warming and anthropogenic disturbances have triggered regeneration failure [27], suppressed seedling growth [28], and simplified stand structure, collectively hindering sustainable forest management. During natural regeneration, the understory light environment represents a critical ecological factor influencing the survival and growth of *P. yunnanensis* seedlings. Shading conditions, arising from alterations in the canopy structure, anthropogenic disturbances, or climate change, frequently modify both the light intensity and the spectral composition in the understory. These modifications subsequently impact seedling photosynthetic efficiency, carbon metabolic processes, and biomass allocation strategies. Field measurements have demonstrated that the photosynthetically active photon flux density (PPFD) in natural forest understories ranges from merely 2 to 18% of full sunlight [29]. This intensity persistently approaches or falls below the light compensation point of *Pinus yunnanensis* seedlings. Given that non-structural carbohydrates (NSCs) serve as critical physiological buffers against environmental stress [30], light intensity gradients in such carbon-limited habitats directly govern the carbon acquisition–consumption balance (photosynthesis vs. respiration), thereby functioning as key threshold determinants for seedling survival [31]. When the PPFD declines below species-specific thresholds, sustained respiratory carbon losses exceed the assimilation gains, ultimately depleting the NSC reserves and triggering carbon-starvation-induced mortality. Seedling age, a key indicator of the ontogenetic stage, significantly influences the carbon storage capacity, metabolic activity, and environmental adaptability. These age-dependent differences may directly shape the response strategies of seedlings to shade stress. Research indicates that seedling age is a key developmental variable driving shifts in NSC allocation strategies. This process manifests distinct stage-specific dynamics under environmental stressors such as drought and shading. During the early developmental stage, carbon coordination among organs predominates, as evidenced by significant positive correlations in the soluble sugar content across the roots, stems, and leaves of *Quercus mongolica* seedlings. This mechanism ensures the efficient translocation of photosynthetic assimilates to functional organs, supporting rapid biomass accumulation [32]. At the intermediate developmental stage, a transition occurs toward inter-organ carbon conversion strategies. Under drought stress, *Robinia pseudoacacia* saplings in this phase convert leaf starch into soluble sugars to enhance the osmotic adjustment, while their root systems exhibit peak activity of antioxidant enzymes (APX, SOD) [33]. At the mature stage, an aboveground carbon prioritization pattern emerges, accompanied by a quantum leap in the carbon reserve capacity. For instance, *Betula ermanii* old-growth seedlings exhibit comparatively smaller NSC declines under severe drought and accelerated recovery post-rehydration, achieved through elevated starch reserves and highly developed root systems [34].

Consequently, investigating the varying patterns of NSC content in different organs of *Pinus yunnanensis* seedlings under shade conditions is essential for elucidating their carbon allocation mechanisms and stress adaptation capacity. Therefore, this study investigated one- and three-year-old *Pinus yunnanensis* seedlings subjected to varying shade levels. We analyzed the response characteristics of non-structural carbohydrates (NSC) and their components (soluble sugars and starch) in the needles, stems, and roots, aiming to elucidate the mechanistic role of seedling age in carbon metabolic adaptations. The findings of this study will enable the development of age-specific carbon starvation threshold models, providing a theoretical basis and technical framework for *P.yunnanensis* seedling regeneration management, understory microenvironment regulation, and precision shading management in plantations.

## 2. Materials and Methods

### 2.1. Study Site

This experiment was conducted at the Arboretum of Southwest Forestry University in Kunming, Yunnan (102°46′ E, 25°03′ N), situated within a subtropical plateau monsoon climate zone at an elevation of 1964 m. The site exhibits mild climatic conditions characterized by a mean annual temperature of 16.5 °C, a mean annual precipitation of 1035 mm, and a mean annual relative humidity of 67%. Within rain-out shelters, the temperature ranged from 18.5 °C to 37 °C, while the relative humidity fluctuated between 22.3% and 48.0%. The seedlings were grown in a substrate comprising a 3:2 (*v*/*v*) mixture of lateritic red soil and humus soil, with the following key properties: a bulk density (soil core method) of 1.001 g·cm^−3^, a field capacity of 22.5%, 3.26 g·kg^−1^ of total carbon, 5.98 g·kg^−1^ of total nitrogen, 0.62 g·kg^−1^ of total phosphorus, and a pH of 7.65.

### 2.2. Plant Material and Experimental Design

*Pinus yunnanensis* seedlings of two age classes (1- and 3-year-old) were sourced from certified superior germplasm (certification No.: Yun R-SS-PY-035-2020) cultivated at Malonghe Forest Farm, Shuangbai County, through Yiliang Garden Nursery. The seedlings were transported to the Arboretum of Southwest Forestry University on 14 March 2024, for pre-transplant acclimatization. On 21 March 2024, transplantation was conducted using plastic pots with the following specifications: For 1-year-old seedlings, the pot dimensions measured 20.5 cm (top diameter) × 14.5 cm (bottom diameter) × 18.5 cm (height) with a target weight of 3 kg, while 3-year-old seedlings were planted in larger pots of 29.5 cm (top) × 23.0 cm (bottom) × 21.0 cm (height) with a 6 kg target weight. All the pots contained single seedlings and were placed on trays. Post-transplantation, the soil moisture was maintained at field capacity to ensure establishment. The experimental area was covered with plastic sheeting to minimize groundwater vapor interference. The seedlings underwent a 2-month acclimatization period under optimal conditions prior to the experimental treatments.

The experiment employed black shade netting to establish five shade treatments with varying canopy layers. To prevent lateral light interference, the periphery of each treatment plot was also enclosed with shade cloth. At noon on clear days, an auto-ranging light meter (LI-250A, Li-Cor, Lincoln, OR, USA) was first used to measure the full-sunlight intensity on open ground; shade houses were then constructed to deliver the target irradiances based on these readings. Five light regimes were created following [35]: full sunlight (CK, 2409–2427 μmol·m^−2^·s^−1^), 80% full sunlight (L1, 1904–1973 μmol·m^−2^·s^−1^), 45% full sunlight (L2, 1025–1105 μmol·m^−2^·s^−1^), 30% full sunlight (L3, 697–777 μmol·m^−2^·s^−1^), and 5% full sunlight (L4, 156–236 μmol·m^−2^·s^−1^). The experiment utilized a completely randomized design featuring ten treatment combinations derived from five light levels (CK- L4) and two seedling ages (one-year-old and three-year-old). Three biological replicates were established per treatment combination, with each replicate comprising ten seedlings. Consequently, 150 one-year-old and 150 three-year-old *Pinus yunnanensis* seedlings were employed in total. The shade treatments began on 21 May 2024. The soil volumetric water content was monitored daily with a soil moisture sensor and maintained at 80 ± 5% of field capacity (corresponding to an actual water content of 36.13–41.38%) by weighing each pot at 17:00 h and replenishing the lost mass with water. Sampling was conducted on 21 August 2024 after 90 days of treatment.

### 2.3. Indicator Measurements

On each sampling date, the seedling height (from ground level to apical bud; precision: 0.1 cm) was measured using a ruler, and the root collar diameter (precision: 0.01 mm) was determined with a vernier caliper. Sampling was conducted for the ten treatment combinations derived from five light levels (CK to L4) and two seedling ages (one-year-old and three-year-old). Five seedlings were randomly selected from each treatment combination, resulting in a total of fifty seedlings across both age groups. The sampling was entirely randomized to eliminate any human bias. The seedlings were harvested using the whole-plant excavation method: plants were removed from their pots, the soil was rinsed off with tap water, and residual moisture was blotted with filter paper followed by absorbent paper. Non-structural carbohydrates (NSCs) were quantified as the sum of the soluble sugars (SS) and starch (ST). Soluble sugars were determined using the anthrone colorimetric assay, while the starch content was measured using the phenol–sulfuric acid method [36]. The concentrations in each organ were calculated against glucose standard curves. The NSC content represents the total of the soluble sugar and starch concentrations.

### 2.4. Statistical Analysis

The statistical analyses encompassed data preprocessing in Microsoft Excel 2016 and visualization in OriginPro 2024 (results are presented as mean ± SD), with hypothesis testing executed in SPSS Statistics 27.0. This included a one-way ANOVA and a two-way ANOVA of the non-structural carbohydrates (NSC) and their components (soluble sugars, starch) across three seedling organs (needles, stems, and roots), followed by Tukey’s HSD post hoc testing (*p* = 0.05) for significance determination and a principal component analysis (PCA) to explore the underlying variability in the dataset.

Independent samples t-tests were used to evaluate age-dependent differences between the seedling cohorts for all the measured parameters. The data were standardized using the SPSS 27.0 software to obtain new data and passed the test of applicability of the data correlation matrix factor analysis. Y1 = Z1 × X1 + Z2 × X2 +… +Z20 × X20, Y2 = Z1 × X1 + Z2 × X2 +… +Z20 × X20. Y = Y1 × principal component 1 variance contribution +Y2 × principal component 2 variance contribution. Y1 is the principal component 1 score, Y2 is the principal component 2 score, Y is the composite score, Z is the coefficient of the principal component scores, and X is the standardized value of growth and physiological indexes.

The phenotypic plasticity index is as follows: PPI = (X_max_ − X_min_)/X_max_, where X_max_ and X_min_ denote the maximum and minimum values of each indicator, respectively.

## 3. Results and Analysis

### 3.1. Effects of Shading on Needle Non-Structural Carbohydrates and Their Components in Pinus yunnanensis Seedlings of Different Ages

**Figure 1:**
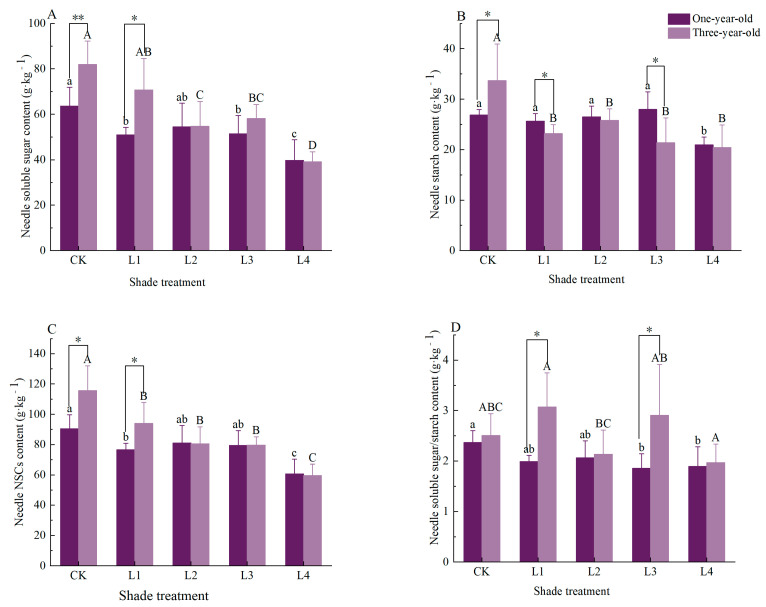
Effects of shading on needle NSCs and their components in *Pinus yunnanensis* seedlings of different ages. Different capital letters on the column indicate that there are significant differences between different shading treatments for three-year-olds (*p* < 0.05), and different lowercase letters indicate that there are significant differences between different shading treatments for one-year-olds (*p* < 0.05). * means significant difference between different ages (*p* < 0.05), ** means extremely significant difference between different ages (*p* < 0.01). Figure (**A**) needle soluble sugar content, Figure (**B**) needle starch content content, Figure (**C**) needle NSC content content, Figure (**D**) needle soluble sugar/starch content. The same notation applies to subsequent figures.

The effects of shading on needle non-structural carbohydrates (NSCs) and their components in *Pinus yunnanensis* seedlings of different ages are presented. For one-year-old seedlings, the needle soluble sugar content under the control (CK) treatment was significantly higher than under the L1, L3, or L4 treatments, with reductions of 19.9%, 19.1%, and 37.6%, respectively, relative to CK (*p* < 0.05). The needle starch content under CK was significantly higher than under L4 (*p* < 0.05), but showed no significant difference compared to L1, L2, or L3. The total NSC content in the needles was significantly lower under L1 and L4 compared to CK, decreasing by 15.3% and 33.0%, respectively (*p* < 0.05). The ratio of soluble sugars to starch under CK was significantly higher than under L3 or L4, with reductions of 21.6% and 20.0%, respectively (*p* < 0.05) (Figure 1).

For three-year-old *Pinus yunnanensis* seedlings, the needle soluble sugar content showed no significant difference between the control (CK) and L1 treatments; however, it was significantly higher under CK than under L2, L3, or L4, with reductions of 33.2%, 29.1%, and 52.3%, respectively, relative to CK (*p* < 0.05). The needle starch content under all shading treatments (L1, L2, L3, and L4) was significantly lower than under CK, decreasing by 31.1%, 23.4%, 36.5%, and 39.4%, respectively (*p* < 0.05). The total needle NSC content was the highest under CK and differed significantly from all the shading treatments (L1–L4). It exhibited progressive reductions with increasing shading intensity, decreasing by 18.7%, 30.4%, 31.2%, and 48.5% relative to CK, respectively (*p* < 0.05). Despite showing no significant differences between CK and the shading treatments, the soluble-sugar-to-starch ratio reached its maximum under the L1 treatment (Figure 1).

A comparative analysis between one-year-old and three-year-old seedlings revealed significant age-dependent responses to shading treatments. Under the CK treatment, the needle soluble sugar content was significantly higher (*p* < 0.01) in three-year-old seedlings compared to one-year-old seedlings, with a 28.7% increase. Conversely, the needle starch content and the total NSC were significantly lower (*p <* 0.05) in one-year-old seedlings, showing reductions of 25.3% and 27.7%, respectively. Under L1 shading, one-year-old seedlings exhibited significantly lower values than three-year-olds for their soluble sugar content (38.8% lower), total NSC (22.6% lower), and soluble-sugar-to-starch ratio (54.0% lower) (*p* < 0.05). Notably, the needle starch content in three-year-olds was significantly reduced under the L1 and L3 treatments compared to one-year-olds, decreasing by 9.0% and 23.6%, respectively (*p* < 0.05). Simultaneously, the soluble-sugar-to-starch ratio under L3 was significantly lower (56.6% reduction) in one-year-old seedlings relative to three-year-olds (*p* < 0.05).

### 3.2. Effects of Shading on Stem Non-Structural Carbohydrates and Their Components in Pinus yunnanensis Seedlings of Different Ages

**Figure 2:**
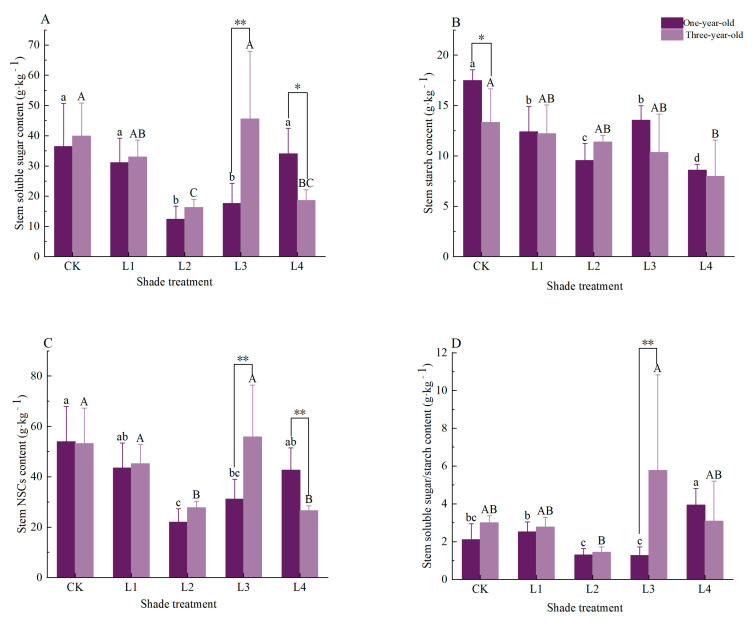
Effects of shading on stem NSCs and their components in *Pinus yunnanensis* seedlings of different ages. Different capital letters on the column indicate that there are significant differences between different shading treatments for three-year-olds (*p* < 0.05), and different lowercase letters indicate that there are significant differences between different shading treatments for one-year-olds (*p* < 0.05). * means significant difference between different ages (*p* < 0.05), ** means extremely significant difference between different ages (*p* < 0.01). Figure (**A**) stem soluble sugar content, Figure (**B**) stem starch content content, Figure (**C**) stem NSC content content, Figure (**D**) stem soluble sugar/starch content.

For one-year-old seedlings (Figure 2), the stem soluble sugar content significantly decreased by 65.9% and 59.8% under the L2 and L3 treatments, respectively, compared to CK (*p* < 0.05). Conversely, the stem starch content was significantly reduced under all the shading treatments (L1-L4) relative to CK, with decreases of 29.1%, 45.4%, 22.5%, and 50.9% (*p* < 0.05). The total stem NSC content similarly showed significant reductions of 59.3% and 42.3% under the L2 and L3 treatments versus CK (*p* < 0.05). Notably, the soluble-sugar-to-starch ratio peaked under the L4 treatment, demonstrating a significant 87.3% increase relative to CK (*p* < 0.05). For three-year-old seedlings (Figure 2), the stem soluble sugar content significantly decreased by 59.1% and 53.3% under the L2 and L4 treatments, respectively, compared to CK (*p* < 0.05). The stem starch content exhibited progressive reductions with increasing shading intensity, culminating in a significant 40.2% decrease under L4 relative to CK (*p* < 0.05). Similarly, the total stem NSC content showed significant decreases of 47.9% and 50.1% under the L2 and L4 treatments versus CK (*p* < 0.05). Although the soluble-sugar-to-starch ratio reached its maximum under the L3 treatment, it showed no significant difference compared to CK.

Under the CK treatment, the starch content in the stems of three-year-old *Pinus yunnanensis* was significantly lower than that of one-year-old seedlings, and was also markedly below 23.7% (*p* < 0.05). Under the L3 treatment, three-year-old *Pinus yunnanensis* seedlings exhibited a significantly higher stem soluble sugar content (↑159.3%) and stem NSC content (↑79.6%) compared to one-year-old seedlings. Furthermore, the soluble-sugar-to-starch ratio in three-year-old seedlings under the L3 treatment was significantly elevated (355.2% higher than the one-year-old counterparts; *p* < 0.01). Under other treatments, no significant differences were observed between one-year-old seedlings and three-year-old plants.

### 3.3. Effects of Shading on Root Non-Structural Carbohydrates and Their Components in Pinus yunnanensis Seedlings of Different Ages

**Figure 3:**
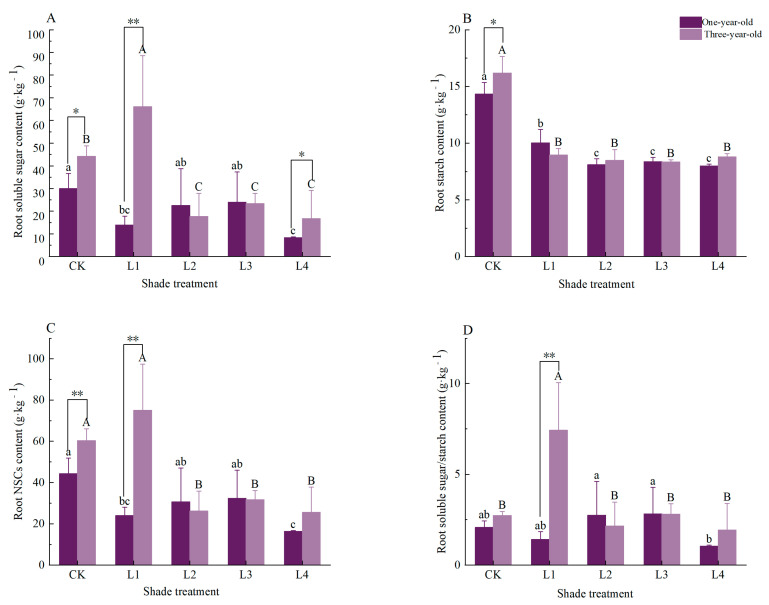
Effects of shading on root NSCs and their components in *Pinus yunnanensis* seedlings of different ages. Different capital letters on the column indicate that there are significant differences between different shading treatments for three-year-olds (*p* < 0.05), and different lowercase letters indicate that there are significant differences between different shading treatments for one-year-olds (*p* < 0.05). * means significant difference between different ages (*p* < 0.05), ** means extremely significant difference between different ages (*p* < 0.01). Figure (**A**) root soluble sugar content, Figure (**B**) root starch content content, Figure (**C**) root NSC content content, Figure (**D**) root soluble sugar/starch content.

For the roots of one-year-old seedlings (Figure 3), the soluble sugar content significantly decreased by 53.5% and 72.1% under the L1 and L4 treatments, respectively, compared to CK (*p* < 0.05). The starch content exhibited significant reductions across all the shading treatments (L1-L4), declining by 30.1%, 43.4%, 41.5%, and 50.2% relative to CK (*p* < 0.05). The total NSC content was significantly lower under the L1 and L4 treatments, showing decreases of 45.9% and 63.1% versus CK (*p <* 0.05). However, the soluble-sugar-to-starch ratio showed no significant differences among any treatment groups. For the roots of three-year-old seedlings (Figure 3), the soluble sugar content peaked under the L1 treatment, showing a significant 49.5% increase relative to CK (*p* < 0.05). In contrast, substantial decreases occurred under the L2 (59.9% lower), L3 (47.1% lower), and L4 (62.1% lower) treatments compared to CK (*p* < 0.05). The starch content was significantly reduced across all the shading treatments (L1-L4), decreasing by 44.7%, 47.5%, 48.4%, and 45.7%, respectively *(p* < 0.05). The total NSC content exhibited significant reductions of 56.6%, 57.7%, and 65.9% under the L2, L3, and L4 treatments versus CK (*p* < 0.05), while reaching its maximum value (75.05 mg·g^−1^) under L1. The soluble-sugar-to-starch ratio showed a pronounced 171.8% increase under L1 compared to CK (*p* < 0.05), though CK displayed no significant differences relative to the other treatments.

A comparative analysis of the root components revealed significant age-dependent variations. Under CK conditions, three-year-old seedlings exhibited a significantly higher root soluble sugar content (47.3% greater) and starch content (12.9% greater) compared to one-year-olds (*p* < 0.05). Notably, the total root NSC content was substantially elevated in three-year-olds, showing a 36.2% increase over one-year-olds (*p* < 0.01). Under L1 shading, three-year-olds demonstrated striking enhancements in their soluble sugar content (373.9% higher), total NSCs (213.1% higher), and soluble-sugar-to-starch ratio (426.0% higher) relative to one-year-olds (*p* < 0.01). No significant differences between the age groups were observed across the other shading treatments.

### 3.4. Analysis of Phenotypic Plasticity in Non-Structural Carbohydrate Response to Shading in Pinus yunnanensis Seedlings of Different Ages

**Figure 4:**
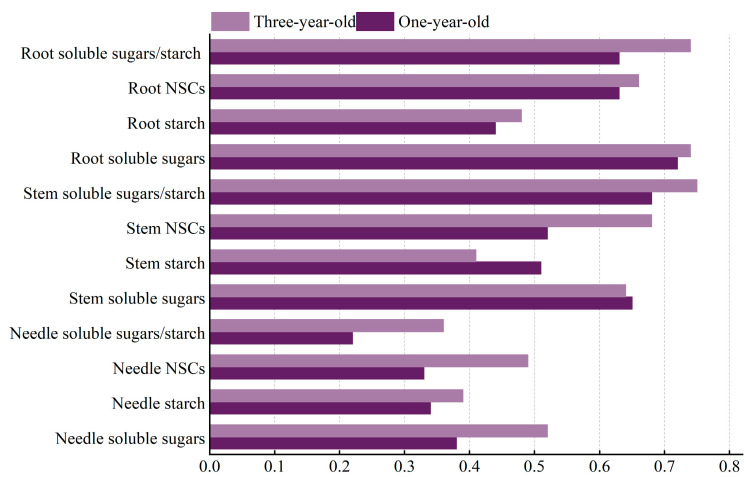
Analysis of phenotypic plasticity in NSC response to shading in *Pinus yunnanensis* seedlings of different ages.

Figure 4 presents the phenotypic plasticity indices (PPIs) of NSCs and their components across organs in *Pinus yunnanensis* seedlings of different ages under shading treatments. Distinct PPI patterns emerged between the age classes: For one-year-old seedlings, the highest plasticity occurred in the root soluble sugars, stem soluble-sugar-to-starch ratio, and stem soluble sugars. Moderately plastic traits included the root soluble-sugar-to-starch ratio, root NSCs, stem NSCs, stem starch, and root starch, while the needle tissues exhibited minimal plasticity. Among three-year-olds, peak plasticity was observed in the stem soluble-sugar-to-starch ratio, root soluble-sugar-to-starch ratio, and root soluble sugars. Intermediate plasticity characterized the stem NSCs, root NSCs, stem soluble sugars, needle soluble sugars, and needle NSCs. The lowest plasticity indices occurred in the root starch, stem starch, needle starch, and needle soluble-sugar-to-starch ratio.

Comparatively, one-year-olds showed a slightly higher plasticity in their stem NSCs and stem starch than three-year-olds, whereas all other PPIs were generally lower in younger seedlings.

### 3.5. Correlation Analysis of Shading Effects on Non-Structural Carbohydrates in Pinus yunnanensis Seedlings of Different Ages

**Figure 5:**
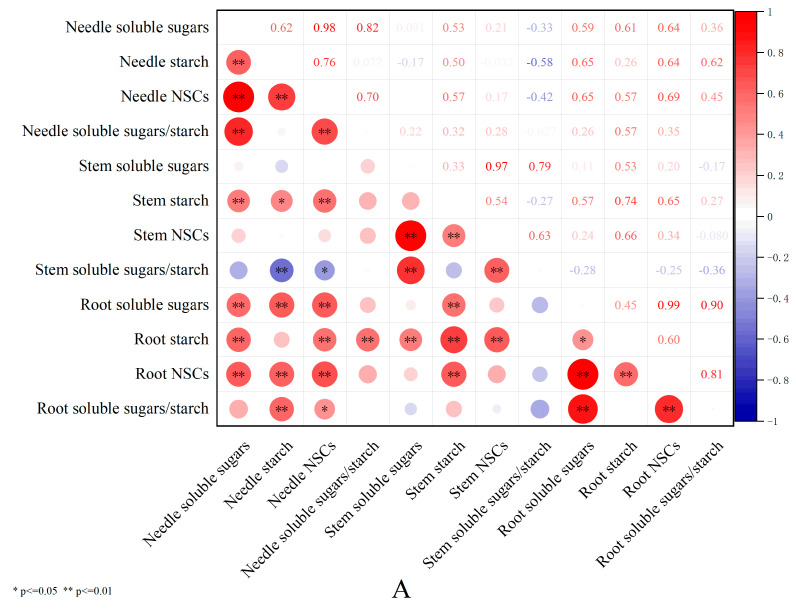

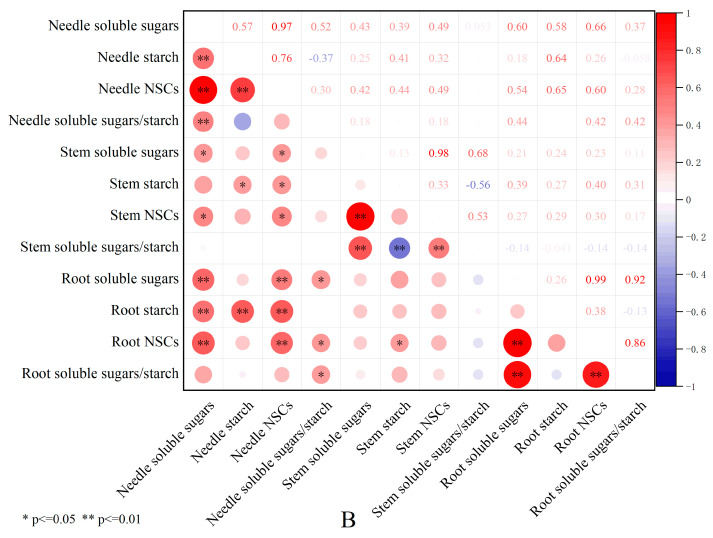
Correlation analysis of shading effects on NSCs in *Pinus yunnanensis* seedlings of different ages. (**A**): One-year-old. (**B**): Three-year-old. Note: * indicates *p <* 0.05, ** indicates *p <* 0.001.

As shown in Figure 5A, the correlation analysis of the shading effects on non-structural carbohydrates (NSCs) in one-year-old *Pinus yunnanensis* seedlings revealed widespread intercorrelations among NSC components across all organs. Among all three organs (roots, stems, and leaves), universally significant positive correlations (*p* < 0.01) were demonstrated between NSCs and their components: soluble sugars, starch, and the soluble-sugar-to-starch ratio. The needle soluble sugars showed highly significant positive correlations (*p* < 0.01) with the root soluble sugars, root starch, root NSCs, and stem starch. The needle starch exhibited highly significant positive correlations with the root soluble sugars, root NSCs, and root soluble-sugar-to-starch ratio (*p* < 0.01), while demonstrating a significant positive correlation with the stem starch (*p* < 0.05). The needle NSCs maintained highly significant positive correlations with the root soluble sugars, root starch, root NSCs, and stem starch (*p* < 0.01), alongside a significant positive correlation with the root soluble-sugar-to-starch ratio (*p* < 0.05). The needle soluble-sugar-to-starch ratio showed a highly significant positive correlation with the root starch (*p* <0.01). The stem soluble sugars, stem starch, and stem NSCs each demonstrated highly significant positive correlations with the root starch (*p* < 0.01). Similarly, the root soluble sugars and root NSCs showed highly significant positive correlations with the stem starch (*p* < 0.01). Crucially, the stem soluble-sugar-to-starch ratio exhibited a highly significant negative correlation with the needle starch (*p* < 0.01) and a significant negative correlation with the needle NSCs (*p* < 0.05).

As shown in Figure 5B, the correlation analysis of the shading effects on non-structural carbohydrates (NSCs) in three-year-old *Pinus yunnanensis* seedlings revealed distinct patterns. Within the needle organs, the total NSCs showed highly significant positive correlations with the needle soluble sugars and needle starch (*p* < 0.01). In the stems and roots, the NSCs maintained highly significant positive correlations with the soluble sugars and soluble-sugar-to-starch ratios (*p* < 0.01). Across organs, the needle soluble sugars and needle NSCs exhibited highly significant positive correlations with the root soluble sugars, root starch, and root NSCs (*p* < 0.01), while showing significant positive correlations with the stem soluble sugars and stem NSCs (*p* < 0.05). The needle starch demonstrated a highly significant positive correlation with the root starch (*p* < 0.01) and a significant positive correlation with the stem starch (*p* < 0.05). The needle soluble-sugar-to-starch ratio showed significant positive correlations with the root soluble sugars, root NSCs, and root soluble-sugar-to-starch ratio (*p* < 0.05). Additionally, the stem starch correlated significantly with the root NSCs (*p* < 0.05), while notably exhibiting a highly significant positive correlation with the stem soluble-sugar-to-starch ratio (*p* < 0.01).

### 3.6. Principal Component Analysis (PCA) of Shading Effects on Non-Structural Carbohydrates in Pinus yunnanensis Seedlings of Different Ages

**Figure 6:**
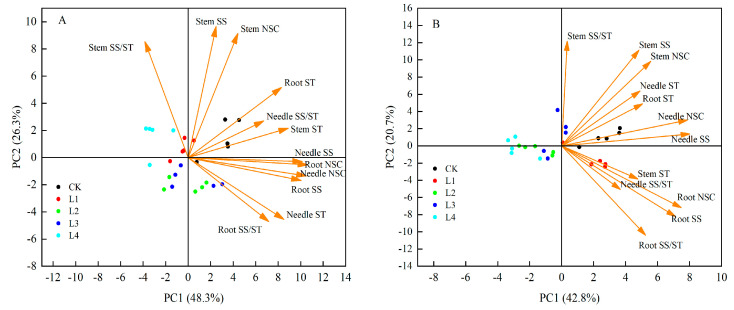
Principal component analysis (PCA) of shading effects on NSCs in *Pinus yunnanensis* seedlings of different ages. (**A**): One-year-old. (**B**): Three-year-old.

The principal component analysis (PCA) of the non-structural carbohydrates (NSCs) and their components in the needles, stems, and roots of *Pinus yunnanensis* seedlings under shading treatments (Figure 6) revealed distinct age-stratified response mechanisms. The cumulative variance explained by the first two principal components reached 74.3% for one-year-old and 63.5% for three-year-old seedlings, indicating that these dimensions effectively capture the age-specific shading responses. In one-year-olds (Figure 6A), PC1 exhibited dominant loadings for the needle soluble sugars, needle NSCs, root soluble sugars, and root NSCs, while PC2 showed the strongest weightings for the stem soluble sugars, stem NSCs, and stem soluble-sugar-to-starch ratio. For three-year-olds (Figure 6B), PC1 was primarily driven by the needle soluble sugars and needle NSCs, whereas PC2 demonstrated maximal weighting for the stem soluble-sugar-to-starch ratio. These patterns indicate that the shading responses in one-year-old seedlings are governed through the leaf–root coordination of soluble sugars and NSC dynamics (PC1), while three-year-olds mediate carbon homeostasis via the stem-based regulation of sugar–starch ratios (PC2).

## 4. Discussion

### 4.1. Organ-Specific Non-Structural Carbohydrate Responses to Shading Stress in Pinus yunnanensis Seedlings of Different Ages

In this study, shading treatments significantly altered the accumulation and partitioning of non-structural carbohydrates (NSCs) in *Pinus yunnanensis* seedlings. We observed progressively declining NSC levels with an increasing shading intensity. Specifically, the needle NSC content decreased by 33% in one-year-old and 48.5% in three-year-old seedlings under severe shading (L4 treatment, Figure 1). Under such high shading conditions, the reduced light availability diminished the carbon assimilation rates, consequently constraining NSC accumulation. These findings align with those of Board and Harville [37], who demonstrated that plants with a high photosynthetic potential cannot fully utilize their photosynthetic apparatus in low-light environments, leading to suppressed carbon fixation. The root NSC responses exhibited distinct strategic patterns across seedling ages. In three-year-old *Pinus yunnanensis*, the root NSC content peaked under L1 shading, with the soluble sugars increasing significantly by 49.5% and the soluble-sugar-to-starch ratio rising by 171.8% (Figure 3). This likely reflects a “root-preferential” carbon allocation strategy, where photosynthates are redirected belowground to sustain root respiration, osmoregulation, and fine root growth. Under the L1 treatment, the carbon investment in the roots enhanced sugar accumulation for osmoprotection while maintaining the starch reserves; this balanced allocation supports seedling establishment in understory environments [21]. However, under intensified shading, the persistent suppression of photosynthetic carbon assimilation shifted the root NSC dynamics from accumulation to depletion, indicating a sufficient carbon influx to meet the dual respiratory and growth demands, ultimately causing passive NSC depletion. This aligns with the “photosynthetic limitation → root NSC deficit” pattern observed by Dai et al. in Robinia pseudoacacia and Platycladus orientalis [38]. Under the L4 treatment, the root NSC content in one-year-old *Pinus yunnanensis* seedlings decreased by 63.1% (Figure 3), with starch exhibiting a significantly greater reduction than soluble sugars. This indicates that, under sustained shading stress, the seedlings prioritize mobilizing stored root starch to maintain basal metabolic functions [23]. When the starch reserves become depleted amid a continuing carbon deficit, the soluble sugars subsequently decline, driving seedlings into carbon starvation [39]. This “root-preferential” carbon allocation strategy in three-year-old seedlings under moderate shade (L1 treatment) aligns with their developmental need to enhance the root carbon reserves for long-term adaptive capacity. Conversely, the continuous decline in the root NSCs with increasing shading intensity in one-year-old seedlings—in the absence of a peak response—reflects the prioritization of immediate metabolic demands over reserve accumulation, further demonstrating fundamental differences in age-specific carbon coordination strategies.

Both one- and three-year-old *Pinus yunnanensis* seedlings exhibited declining trends in their stem NSC content under L1 and L2 shading (Figure 2). Under mild shade, seedlings likely prioritize carbon allocation to the roots and needles—supporting critical functions like osmoregulation and photosynthetic recovery—while the stems, as non-essential organs, receive a lower allocation priority. Concurrently, the stems neither cease growth entirely nor obtain a sufficient carbon supply, becoming “sacrificial organs” that experience a carbon imbalance. This supply–demand disparity significantly depletes the stem NSCs, inducing mild “carbon starvation,” consistent with Yan et al. [40]. As the shading intensified, the stem NSCs increased in one-year-olds, recovering to the control (CK) levels under L4 (Figure 2). The three-year-olds reached peak stem NSCs under L3, aligning with McDowell’s “growth cessation-carbon enrichment” hypothesis: under extreme low light, xylem growth essentially halts, reducing the structural carbon demand in stems [39]. This creates a “carbon demand gap” while the root–stem reserve starch undergoes passive hydrolysis, with photosynthates refluxed via the phloem to the stems. Consequently, the assimilates accumulate in the stem parenchyma cells, passively elevating the NSC concentration. This reflects a broader low-light tolerance in stem carbon homeostasis regulation with seedling age. Collectively, these mechanisms reveal physiological tactics for maintaining stem carbon homeostasis through structure–metabolism tradeoffs under carbon limitation.

### 4.2. Organ-Specific Coordinated Resource Allocation Strategies in Pinus yunnanensis Seedlings of Different Ages Under Shading Stress

Under shading stress, *Pinus yunnanensis* seedlings exhibited a significant age-dependent divergence in carbon coordination strategies across the root, stem, and needle organs, transitioning from localized inter-organ coordination to systemic homeostasis with increasing age. The one-year-old seedlings employed a “leaf–root emergency coordination” strategy: Under severe shading (L4 treatment), the needle NSCs decreased by 33% relative to CK, with the soluble sugars declining more markedly (37.6%) than starch (22.1%) (Figure 1), indicating the prioritized consumption of soluble sugars for immediate metabolic maintenance during abrupt photosynthetic carbon reduction. Simultaneously, the root NSCs dropped by 63.1% with significantly greater starch depletion (44.2%) than soluble sugar reduction (72.1%) (Figure 3), reflecting accelerated starch hydrolysis to replenish soluble sugars for osmoregulation and respiratory demands. The phenotypic plasticity analysis revealed elevated plasticity indices for the root soluble sugars and root NSCs (Figure 4), confirming roots as the primary responsive organs in one-year-old seedlings under shading stress. This aligns with Landhäusser and Adams’ conclusion regarding the “prioritized mobilization of root starch to maintain aboveground function in saplings under carbon limitation” [41]. The correlation analysis further demonstrated highly significant positive relationships (*p* < 0.01) between the needle NSCs and the root NSCs, root soluble sugars, and root starch, whereas no significant associations existed with the stem NSCs (Figure 5A). The principal component analysis showed the needle soluble sugars and root soluble sugars as dominant weighting factors in PC1, with the stem soluble sugars contributing minimally to PC2 (Figure 6A). Collectively, these findings indicate a concentrated carbon flux along the leaf–root axis in one-year-old seedlings, facilitating short-term adaptive responses through immediate inter-organ carbon translocation without establishing synergistic coordination with stems. Three-year-old *Pinus yunnanensis* seedlings developed a “root–stem–needle closed-loop homeostatic regulation” strategy. Under severe shading (L4 treatment), their needle non-structural carbohydrate (NSC) content decreased more substantially (48.5%) than in one-year-old seedlings, with parallel reductions in soluble sugars (52.3%) and starch (39.4%) (Figure 1). This reflects a heightened dependence of the carbon assimilation capacity on a strong light intensity as the seedlings age. Conversely, under moderate shade (L1 treatment), the roots exhibited active carbon storage traits: the root NSCs peaked, with the soluble sugars increasing significantly by 49.5% and the soluble-sugar-to-starch ratio rising sharply by 171.8% (Figure 3). This demonstrates a “root-preferential” carbon allocation strategy [21], aligning with Chen et al.’s conclusion that “roots function as active carbon sinks under moderate shading” [42]. The correlation analysis revealed that the needle NSCs in three-year-old seedlings showed highly significant positive correlations (*p* < 0.01) with the root NSCs, root soluble sugars, and root starch, alongside a significant positive correlation (*p* < 0.05) with the stem NSCs (Figure 5B). This indicates an expansion of carbon flux across the integrated leaf–root–stem system. The principal component analysis identified the stem soluble-sugar-to-starch ratio as the dominant weighting factor in principal component 2 (PC2) (Figure 6B). Combined with the minimal decline in the stem NSCs during shading, this suggests that the highly lignified stems—which exhibit reduced structural growth demands—function as stable carbon reservoirs. These stems form a “root–stem carbon buffer system” with the roots, consistent with Sala et al.’s mechanism of “organ carbon demand gradients regulating carbon allocation.” Notably, under the L3 treatment, the stem soluble-sugar-to-starch ratio surged by 355.2%. This confirms the stem’s participation in systemic regulation through carbon form interconversion. Ultimately, this establishes a closed-loop “root-storage → stem-buffering → leaf-supply” carbon regulation network.

In summary, the carbon coordination strategies of *Pinus yunnanensis* seedlings exhibit an age-dependent progression from a dichotomous to a tripartite regulatory pattern: one-year-old seedlings rely on localized “leaf–root coordination” for an immediate stress response, while three-year-old seedlings establish systemic homeostasis through integrated “root–stem–needle synergy.” This age-dependent intensification of organ coordination fundamentally represents a strategic shift in the carbon economy—from prioritizing immediate survival toward ensuring long-term robustness—which ultimately determines the distinct shade tolerance thresholds and low-light adaptation capacities observed across seedling age classes. Collectively, these findings provide critical empirical evidence for deciphering the age-dependent nature of plant carbon coordination strategies while establishing a theoretical foundation for age-specific shading management practices.

### 4.3. Ontogenetic Shade Thresholds Informing Regeneration Management for Pinus yunnanensis Plantations

This study reveals the adaptation thresholds of *Pinus yunnanensis* seedlings of different ages under shading stress. One-year-old *P. yunnanensis* seedlings experienced a 19.9% decrease in their needle NSCs under the L1 treatment (Figure 1) and a 63.1% reduction in their root NSCs under the L4 treatment (Figure 3), indicating a fragile carbon balance and a high risk of carbon starvation. In contrast, three-year-old seedlings showed a 49.5% increase in their root carbon reserves under the L1 treatment (Figure 3), with the root NSCs reaching their peak (75.05 kg·g^−1^). Moreover, under the L3 treatment, the ratio of soluble sugars to starch in the stem increased by 355.2% (Figure 2), achieving carbon enrichment. This is consistent with the findings of Sala et al., who reported that, under moderate shading, more mature seedlings allocate carbon resources to storage organs such as roots and stems, creating a “carbon reservoir” [25]. Therefore, in the early stages of artificial forest regeneration (e.g., 1–2 years after sowing), it is necessary to thin the upper canopy or control the cover of shrubs and grasses to maintain a PPFD ≥80% of full sunlight in the understory, avoiding carbon imbalances and death [41]. At the same time, it is important to avoid heavy shading caused by adjacent tall vegetation or the excessive retention of the upper canopy. For one-year-old seedlings, the canopy closure should be controlled to ≤20% (i.e., ≥80% full sunlight); for three-year-old seedlings; moderate shading (canopy closure of 20–55%) can be provided to promote root development. The principal component analysis (Figure 6) indicates that three-year-old seedlings achieve system homeostasis through “root–stem–leaf” closed-loop regulation, while one-year-old seedlings rely on a “leaf–root” binary linkage. This suggests that, in the establishment of mixed forests, a stepped shading structure can be designed, with open spaces (high-light areas) retained in the one-year-old seedling zones and three-year-old seedling zones configured in the subcanopy (moderate shading areas) to simulate the light gradient in natural regeneration [42]. This can coordinate the carbon allocation efficiency of the community while enhancing the overall carbon storage of the forest stand. Under the L4 treatment, both seedling ages experienced a root starch reduction of >40% (Figure 1, Figure 2 and Figure 3), triggering the risk of carbon starvation. This is consistent with the field observations of Nelson et al., who reported that the PPFD under natural forest canopies is often below the light compensation point (< 18% of full sunlight), leading to the failure of *P. yunnanensis* regeneration [29]. Therefore, regular canopy thinning to maintain the PPFD at 30% in the understory is essential to ensure the dynamic balance of the NSC pool.

In summary, implementing age-differentiated shading management can significantly reduce the early mortality rate of one-year-old seedlings while providing an optimized environment for three-year-old seedlings to promote carbon reserve accumulation and root growth. The enhanced root carbon storage and the established “three-dimensional carbon insurance network” under moderate shading for three-year-old seedlings mean that seedlings with robust root systems and ample carbon reserves are more likely to develop into sturdy trees, thereby enhancing the entire forest stand’s resistance and resilience to disturbances. Optimized shading management can improve the success rate of artificial forest regeneration, ensuring that a sufficient number of healthy seedlings enter the next growth stage, thus constructing a forest stand with a rational age structure and uniform spatial distribution, and avoiding large canopy gaps or homogeneous forest structure caused by regeneration failure.

## 5. Conclusions

This study elucidated the age-specific mechanisms of non-structural carbohydrate (NSC) responses to shading stress in *Pinus yunnanensis* seedlings, revealing ontogenetic patterns in organ-specific carbon coordination strategies. The shading intensity drives the NSC dynamics by modulating the carbon-assimilation–respiration balance, confirming the disruption of carbon homeostasis under low-light conditions. Age determines the evolutionary pathways of carbon coordination: one-year-old seedlings exhibit “needle–root source–sink reallocation” characterized by preferential root starch consumption for immediate survival, reflecting a binary adaptation to short-term stress. In contrast, three-year-old seedlings develop a “root–stem–leaf closed-loop homeostasis regulation” mechanism, forming a three-dimensional carbon insurance network through cross-organ coordination that significantly enhances their shade tolerance and resilience. We demonstrate an ontogenetic progression in the carbon strategies, from emergency consumption (age 1) to systemic homeostasis (age 3), with organ coordination expanding from localized to pan-organismic levels. This provides novel insights into the age-dependent carbon economy tactics of plants. For the silvicultural management of *P. yunnanensis* regeneration, one-year-old seedlings require ≥80% full sunlight to prevent a carbon imbalance, while three-year-old seedlings optimize their root carbon reserves under 45–80% full sunlight. These light thresholds offer quantitative guidelines for precision light environment management and the ecological restoration of degenerated forests in Southwest China. Future research should address multifactorial interactions by (1) implementing combined shading and water stress treatments to elucidate seedling carbon allocation strategies and physiological responses under compound stress and (2) establishing long-term monitoring plots in natural forest stands to track age-specific carbon metabolism dynamics within plant communities, including interactions with neighboring vegetation and soil microbiomes. These approaches will provide critical insights for evidence-based forest conservation and management.

## Data Availability

The data will be made available upon request.
